# Regulation of anti-tumor immunity by metal ion in the tumor microenvironment

**DOI:** 10.3389/fimmu.2024.1379365

**Published:** 2024-06-10

**Authors:** Yaoxin Gao, Shasha Liu, Yifan Huang, Feng Li, Yi Zhang

**Affiliations:** ^1^ Biotherapy Center & Cancer Center, the First Affiliated Hospital of Zhengzhou University, Zhengzhou, China; ^2^ School of Life Sciences, Zhengzhou University, Zhengzhou, China; ^3^ State Key Laboratory of Esophageal Cancer Prevention & Treatment, Zhengzhou University, Zhengzhou, China; ^4^ School of Public Health, Zhengzhou University, Zhengzhou, China

**Keywords:** metal ions, tumor microenvironment, tumor immunotherapy, nanomedicines, T cells

## Abstract

Metal ions play an essential role in regulating the functions of immune cells by transmitting intracellular and extracellular signals in tumor microenvironment (TME). Among these immune cells, we focused on the impact of metal ions on T cells because they can recognize and kill cancer cells and play an important role in immune-based cancer treatment. Metal ions are often used in nanomedicines for tumor immunotherapy. In this review, we discuss seven metal ions related to anti-tumor immunity, elucidate their roles in immunotherapy, and provide novel insights into tumor immunotherapy and clinical applications.

## 1 Introduction

Cancer is a multifaceted disease characterized with abnormal proliferation of malignant cells. High morbidity and mortality rates as well as restricted treatment options pose a serious threat to public health. Consequently, innovative cancer treatments, such as immunotherapy and photothermal therapy, are emerging in clinical settings and trials (1). Cancer immunotherapy has been successful because of its ability to mobilize immune activation and memory, which results in the rapid and effective control of tumor growth and prevention of recurrence (2). The complex tumor microenvironment (TME) is the main reason for limiting immunotherapy, including immunosuppressive cytokines and cells, such as transforming growth factor-beta (TGF-β), interleukin (IL-10), regulatory T cells (Tregs) and myeloid-derived suppressor cells (MDSCs) (3, 4). With continuous research on immunomodulatory factors, the role of metal ions in tumor immunotherapy has gradually been emphasized (5).

Ion signals are transmitted by various transcellular and intracellular signaling cascades that regulate immunological memory and immune responses. Different metal ions play various roles in signal transmission, including calcium (Ca^2+^), zinc (Zn^2+^), manganese (Mn^2+^), magnesium (Mg^2+^), potassium (K^+^), iron (Fe^2+/3+^), and copper (Cu^+/2+^) (6). Cell membrane is the main barrier limiting free penetration in the process of metal ion transport. The transport of metal ions through active means results in the establishment of concentrations and electric gradients across the membranes. Various integral pore-forming membrane proteins facilitate this process by enabling the movement of ions across membranes, allowing the conversion of different signals through alterations in ion permeability. Currently, nanotechnology provides new methods for clinical tumor immunotherapy and has more advantages than traditional immunotherapy nanocarriers. Nanometallic materials have garnered attention as a promising delivery medium due to their nanocrystalline strengthening, high photoabsorptivity, heightened surface energy, and unique single magnetic domain characteristics (7). Metal ions are important components of nanoparticles to improve their anti-tumor effects (8, 9). Because metal-based nanomaterials are comparable in size to deoxyribonucleic acid (DNA), proteins, viruses, and biomolecules, there are distinctive interactions that occur between metal-based nanoparticles and proteins found in the matrix (10). Additionally, metal-based nanomaterials have the ability to enter the bloodstream and penetrate the TME, enabling them to be absorbed and transported by tumor cells. Crucially, nanomaterials of the appropriate size exhibit a unique enhanced permeability and retention (EPR) effect in tumor sites, significantly enhancing their utilization rate and tumor treatment efficacy (11). In this review, we discuss the role played by different metal ions in anti-tumor immunotherapy and how to use metal ions to improve the efficacy of tumor immunotherapy.

## 2 Metal ions in anti-cancer immunity

### 2.1 Calcium

Ca^2+^ is the most abundant metal ion involved in the proliferation and cycling of immune cells. Currently, Ca^2+^ is the most studied metal ion and widely recognized as a second messenger in immune responses (12). This section will discuss the transport and effect of Ca^2+^ in lymphocytes.

**Figure 1:**
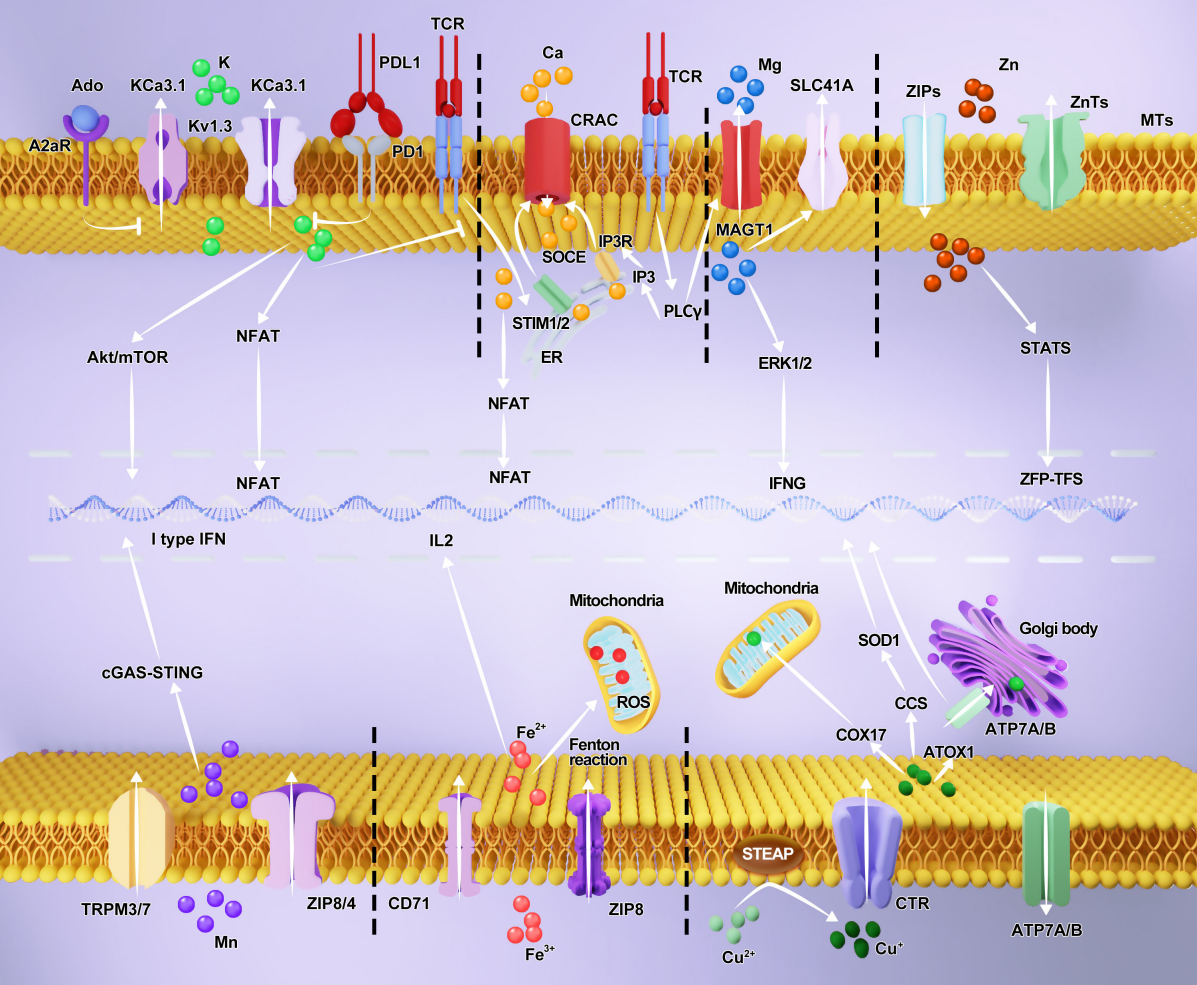
Role of metal ions in T cells. Various metal ion channels or transporters are present on the surface of T cells. They are mainly used to maintain the entry and exit of metal ions. In addition, some metal ions or transporters can regulate the transport of metal ions within the cytoplasm to various organelles and nuclei, thereby altering the function of related organelles or the transcription of target genes, thus regulating the cell function.

The concentration gradient of Ca^2+^ is involved in release and storage and dependent on Ca^2+^ release-activated Ca^2+^ (CRAC) channels, transient receptor potential (TRP) channels, P2X and Ca_v_ channels. After the engagement of immunoreceptors, the mitogenic signal emanating from the IS induces the breakdown of PIP2 to inositol trisphosphate (IP3) binding to the IP3 receptors resulting in the release of Ca^2+^ from the endoplasmic reticulum (ER), and activates stromal interaction molecules (STIM1/2) to bind to the N- and C-termini of ORAI1 which form the subunit of the CRAC channel (13). The CRAC channel exhibits a notable preference for Ca^2+^ and possesses remarkably low single-channel conductance (14, 15). The influx of Ca^2+^ into intracellular organelles is mediated by store-operated Ca^2+^ entry (SOCE) (16, 17) (**Figure 1**).

T cells activated by tumor antigens in an MHC-I-restricted manner begin to proliferate and release cytokines. During this progression, signal transduction considerably relies on key receptors and signaling molecules, including evocation and termination (18). IP3 binds to the IP3 receptor, resulting in Ca^2+^ influx to activate the calcineurin-nuclear factor of activated T (NFAT) pathway, which enhances the cellular processes, for example, polarization, proliferation, and cytokines release including interleukin 2 (IL-2), interferon-γ (IFNγ), tumor necrosis factor (TNF), perforin, and granzyme (19, 20). Abolishment of humanT cell function is caused by deletion of STIM1 or ORAI1 to inhibit Ca^2+^ influx *in vitro* (21). Functional CRAC channels and elevated levels of cytoplasmic Ca^2+^ may activate leukocyte function-associated antigen 1 (LFA-1), contributing to the adhesion of cytotoxic lymphocytes (CTLs) to the target cells both *in vitro* and *in vivo* (22–24). Co-receptor CD5 co-cross-linking with the TCR-CD3 complex down-regulated the TCR-CD3-increased Ca^2+^ mobilization to inhibit T cell function (25). Programmed death-1 (PD1) blockade increases Ca^2+^ signaling to enhance the migratory ability of CTLs in neck cancer (26). P2X, a type of Ca^2+^ channels, enhances T cell function and T cell receptor (TCR) signaling both in human and mouse model (27, 28). TRP and Ca_v_ channels promote rodent and human T cells differentiation and proliferation (29–32) (**Table 1**).

**Table 1:** Role in lymphocytes function.

Metal ion	Role in lymphocytes function					Refs.
	T	DC	NK	Macrophage	Neutrophils	
Ca	T cells function, differentiation, proliferation	Maturation, Antigen presentation	Activation	Polarization and Phagocytosis	Unknown role	(18, 19, 26, 33–36)
Zn	T cells function, differentiation, proliferation	Maturation	Activation, Differentiation	Activation	Recruitment, Chemotaxis	(37–41)
Mn	T cells function, differentiation, proliferation	Maturation	Activation, Infiltration	Polarization, Antigen presentation	Unknown role	(42–45)
Mg	T cells function, proliferation, infiltration	Unknown role	Activation	Unknown role	Unknown role	(46–48)
K	T cells function, differentiation	Unknown role	Unknown role	Polarization	Unknown role	(49–52)
Fe	T cells function	Unknown role	Activation	Polarization	Infiltration	(53–55)
Cu	T cells function, Infiltration	Unknown role	Infiltration	Unknown role	Superoxide anion production	(56–59)

Tregs protect organisms from autoimmune reactions by maintaining T cell tolerance (60). Inhibition or depletion of Tregs enhances the efficacy of cancer immunotherapy (33). Deficiency of either STIM1 or STIM2 in T cells leads to differentiation to Treg, Th1and Th17 cells suggesting that STIM is vital for their development and function (34).

**Figure 2:**
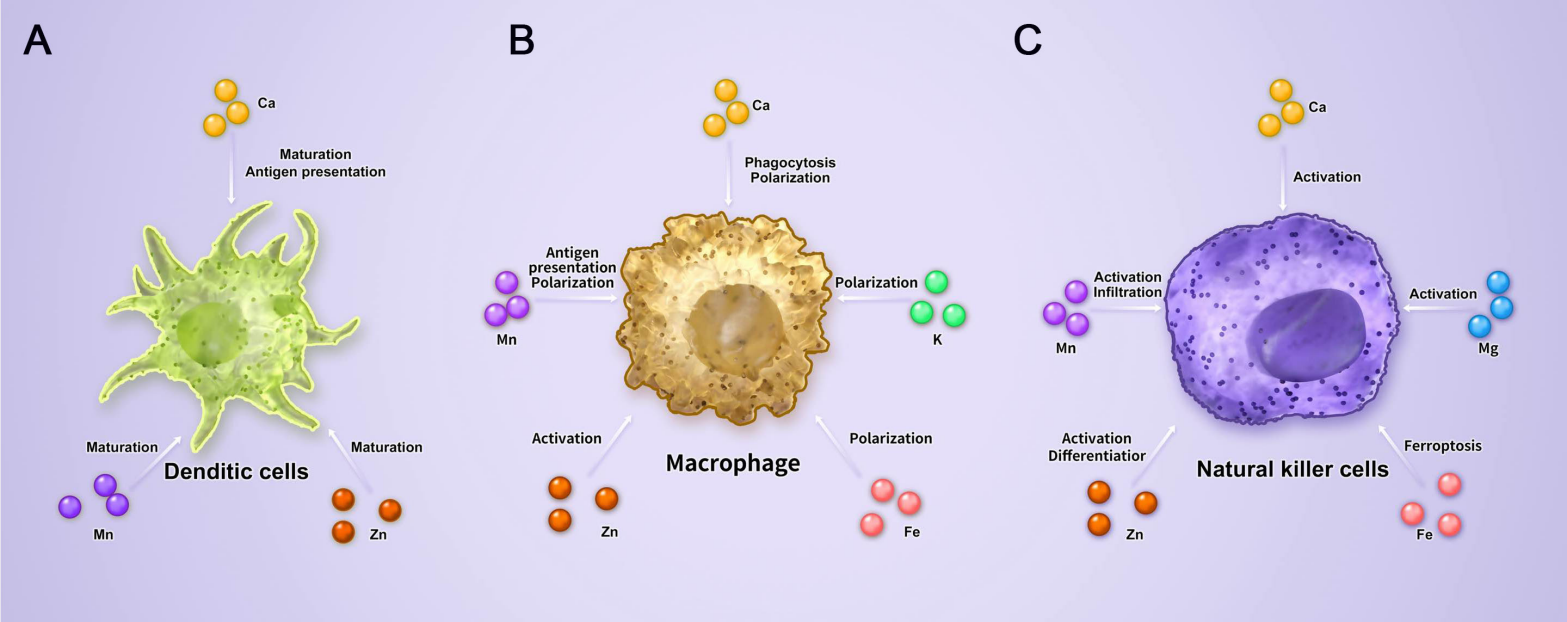
Role of metal ions in dendritic cells, macrophages, and natural killer cells. **(A)** The influence of various metal ions on DCs, the main antigen-presenting cells, is mainly reflected in antigen presentation and maturation. **(B)** Macrophages have both phagocytic and antigen presenting functions, as well as immunosuppressive effects. Therefore, the influence of metal ions on macrophages is mainly reflected in activation, antigen presentation, and polarization. **(C)** NK cells belong to innate immune cells and have strong anti-tumor effects. The main impact of metal ions on NK cells is to regulate their differentiation, activation, and infiltration.

Natural killer (NK) cells originate from bone marrow stem cells, undergo two differentiation and maturation pathways in the bone marrow and thymus, and are widely distributed in the main organs. NK cells selectively kill target cells, such as viral infections or tumors, and play an important role in anti-tumor immunity (35, 36, 61, 62). The ablation of either RAI1 or STIM1 in NK cells severely impairs defective store-operated Ca^2+^ entry but not cytotoxic granule polarization (63, 64). After interaction with target cells, the release of cytokines, such as TNF-α, granulocyte-macrophage colony stimulating factor (GM-CSF), and interferons, by NK cells is increased via the calcineurin-NFAT-dependent pathway (65, 66). Increasing the intracellular Ca^2+^ concentration of dendritic cells (DCs) boosts autophagy and promotes antigen presentation (67). High intracellular Ca^2+^ levels inhibit the formation of WIP/WASP droplets, allowing PKC-θ to easily access and phosphorylate WIP, thereby increasing the actin polarization of macrophages and phagocytosis of tumor cells (68) (**Figure 2**).

Ca^2+^ can bind directly to anionic phospholipids, acting as a regulator for membrane protein functionality. A pH-responsive calcium carbonate nanoparticle (CaCO3/CAI@Liposome, CCL) was prepared by loading calcium carbonate nanoparticles with CAIX inhibitors (CAI) and coating with liposomes, which can accurately regulate the content of Ca^2+^ and pH in and out of tumor cells to promote DCs maturation and macrophage polarization towards the anti-tumor M1 type (69). Given the positive role of Ca^2+^ in immune cells, novel methods should be explored to utilize Ca to improve its anti-tumor effects.

### 2.2 Zinc

Zn^2+^ is a trace metal essential for several proteins and plays a vital role in cell proliferation, differentiation, oxidative stress, immune responses, and apoptosis by conjugating to zinc-binding proteins (37, 70, 71). Moreover, Zn^2+^ participates in various pathophysiological processes, including growth retardation, cognitive disorders, cardiovascular diseases, and cancer (72).

Zn^2+^ homeostasis is tightly regulated by two families of Zn^2+^ transporters: ZnTs and ZIPs (72). ZnTs contain10 members contributing to the export of Zn^2+^ from the intracellular space. ZIPs have 14 members that facilitate an increase in the cytosolic concentration of Zn^2+^ (73). Metal regulatory transcription factor 1 (MTF-1) is sensitive to Zn^2+^ and activated via cysteine2-histidine2 (Cys2-His2) Zn fingers binding to DNA to mediate the expression of ZnT1, ZnT2, and metallothioneins (MTs), which regulate the storage and release of intracellular Zn^2+^ (74, 75) (**Figure 1**).

In the T cell response, the MAPK, PI3K/Akt, and STAT3 pathways are the main signal target transducers of Zn^2+^ which regulate the expression of genes involved in differentiation, survival, proliferation, and apoptosis. ZIP6 expression is enhanced by the activation of the STAT3 signaling pathway to mediate cell migration (38, 39, 76). In sickle cell anemia, Zn^2+^ deficiency decreases CD73, a marker of cytotoxic T cells, resulting in suppressing T cell differentiation and maturation (77, 78). In addition, interaction between the domain of CD4 or CD8α with p56^lck^ is also controlled by Zn^2+^ in regulating T cell activation and differentiation *in vitro* (79). Zap70, a TCR signaling downstream molecule, is phosphorylated through activating Zn^2+^ regulated kinase to sustain proliferation and activation in mouse spleen and thymus cells, as well as human Jurkat T cells. Moreover, Zn^2+^-dependent TCR signal amplification induces IL-2 receptor expression, leading to proliferation of activated T cells (76). In contrast, ZIP6 deletion impairs activation marker expression and cytokine production (80–82). Additionally, SLC39A6 indirectly promotes cell proliferation and cytokine production via TCR activation (83). Moreover, MTs and zinc-finger transcription factors are increased to regulate tumor infiltrating lymphocytes (TILs) in mouse model (40). TILs up-regulate GATA-3 and IKZF2 to mediate Zn^2+^ homeostasis inducing T-cell differentiation and exhaustion in the B16F10 murine melanoma model (84). The balance of T helper cell differentiation is altered by Zn^2+^ signaling. Zn^2+^ depletion decreases IFNγ and T-bet essential for Th1 differentiation in Con-A stimulated HUT-78 cells (41, 84).. TH17 and Treg cells formation is mediated by Zn^2+^ supplementation which remits the T cell-mediated allogeneic immune responses in the human mixed lymphocyte reaction (85, 86). GLI-similar 1 (GLIS1), a zinc finger protein, causes CD8^+^ T cell exhaustion via the SGK1-STAT3-PD1 pathway in hepatocellular carcinoma (HCC) (87). Zinc finger protein 64 (ZFP64) is a transcription factor for cytotoxic T-lymphocyte-associated protein 4 (CTLA-4) and anti- PD-1 in esophageal cancer (88, 89) (**Table 1**).

In addition to affecting T cells, Zn^2+^ can also regulate other immune cells. In mature DCs, the importers ZIP6/10 are decreased, whereas ZnT1/4/6 and MTs are increased to reduce intracellular Zn^2+^ (90). Unlike in DCs, an increase of Zn^2+^ activates macrophages and the recruitment of neutrophils (91). In NK cells, Zn^2+^ promotes the lytic activity and differentiation (92) (**Figure 2**).

Zn^2+^ is essential for innate and adaptive anti-tumor immunity leading to an increase of Zn^2+^-based nanometallic materials. ZnS@BSA (bovine serum albumin), a new zinc-based nanocluster, enhances the infiltration of CD8^+^ T cells and DCs via cGAS-STING signals in HCC (93). Moreover, a Zn-metal-organic skeleton vaccine (ZPM@OVA-CpG) achieved the targeted release of Zn^2+^ in DCs and the TME under acidic conditions. The vaccine actively promoted DCs maturation and antigen cross-presentation via cGAS-STING signaling, resulting in the activation of CD8^+^ T cells (94). Weichselbaum et al. investigated ZnCDA, a powerful tumor-targeting STING agonist, which enhances tumor accumulation by destroying endothelial cells in the tumor vascular system. ZnCDA preferentially targets tumor-associated macrophages to regulate antigen processing and presentation and subsequently triggers anti-tumor T cell responses. ZnCDA reinvigorates the anti-tumor activity of radiation therapy and immune checkpoint inhibitors in immunologically “cold” pancreatic and glioma tumor models (95). Based on the effects on different immune cells, Zn^2+^ is a double-edged sword. Therefore, these factors should be carefully considered before using Zn^2+^.

### 2.3 Manganese

Mn^2+^ is regarded as a second messenger in mammalian tissues and as an essential metal for intracellular processes, such as energy production, bone growth, reactive oxygen species (ROS) generation, and immune response (42, 96). The mobilization and distribution of Mn^2+^ is precisely mediated through gamma globulin, albumin, and Mn-transferrin complex. Mn^2+^ plays a key role in the activation of various enzymes, including glutamine synthetase (GS), isomerases, pyruvate carboxylase, arginase (ARG), lyases, and hydrolases (43, 44).

Usually, intracellular Mn^2+^ is stored in resting organelles, such as the mitochondria or Golgi apparatus. However, damage to mitochondrial activity and DNA can result in the presence of a large amount of intracellular Mn^2+^ (45, 97). Unlike Ca^2+^ and Zn^2+^, intracellular Mn^2+^ is transported using various transporters, such as divalent metal transporter A (DMT1), calcium-based proteins such as TRPM3/7, and metal-dependent proteins such as ZIP8/14 and ZnT10 (42, 98–100) (**Figure 1**).

The effect of Mn^2+^ on T cells is mainly reflected by an increase in the number, differentiation, and cytokine release of TILs by inducing type I interferon production in a murine orthotopic HCC model (101–103). Moreover, Mn^2+^ deficiency leads to a reduction of macrophage antigen presentation and DCs maturation, thereby mediating murine CD8^+^ T cell activation *in vivo* (104). Furthermore, Mn^2+^ treatment substantially increases the infiltration of murine NK cells and the release of cytokines to reduce tumor growth (105, 106). Mn^2+^ can change macrophage (M2) polarization to macrophage (M1) with anti-tumor effects (107). Mechanistically, the Mn^2+^-dependent cGAS-STING pathway plays a remarkable role in T and NK cell functions and DCs maturation. It was reported that NK cell activity increased by producing type I IFNs after adding MnCl_2_ (103). In addition, Mn^2+^ improved the clinical efficacy of PD-1/PD-L1 therapy (101, 108). Taken together, Mn^2+^ serves a vital function in the cross-presentation of immune cells, such as DCs, TILs, and NK cells (**Figure 2**).

Manganese oxide (MnO_2_) nanomaterials are typically used as enhancers to increase the immunotherapy efficiency of photothermal agents by catalyzing the conversion of H_2_O_2_ into O_2_ (109). Mn@CaCO3/ICG nanoparticles serve as vectors for loading small interfering RNA (siRNA) targeting PD-L1 (109). Hou et al. designed MnO nanoparticles combined with the tumor-homing peptide iRGD (CRGDKGPD) to effectively activate the cGAS-STING signaling pathway. Manganese also assisted α-PD-1 to inhibit tumor growth and metastasis (110). Therefore, Mn^2+^ is a potential therapeutic adjuvant for the treatment of tumors.

### 2.4 Magnesium

Mg^2+^ is the second-most abundant metal ion (111, 112). Mg^2+^ is normally complexed with ATP or ADP or acts as a cofactor, which is essential in the regulation of biological activities, such as the cell cycle, apoptosis, division, and differentiation (46, 113), as well as biochemical processes, including oxidative phosphorylation, glycolysis, DNA repair, metabolism, and synthesis (114–116).

Mg^2+^ distribution is different among the extracellular and intracellular spaces, which determines its various functions. Extracellular Mg^2+^ binds to plasma proteins to transmit activating or inhibitory signals, whereas intracellular Mg^2+^ interacts with nucleotides, nucleic acids, or ATP to mediate biochemical processes (117). The Mg^2+^ transport is tightly regulated by intake and excretion via Mg transporter 1 (MAGT1), TRP cation channel subfamily M member 7 (TRPM7), SLC41A1, and Na^+^ exchange; MAGT1 is a highly selective Mg^2+^ channel (47, 118, 119) (**Figure 1**).

Mg^2+^ is regarded as a second messenger that regulates proliferation, development, and function through MAGT1, TRPM7, and SLC41A1 transporters in T cells (48, 120),. After TCR stimulation or PLCγ1 activation, Mg^2+^ and Ca^2+^ enter T cells in an MAGT1-dependent manner (121). The deletion of MAGT1 decreases the capacity response to TCR and Ca^2+^ entry into human T cells *in vitro* (48, 122). In contrast, reducing the concentration of Mg^2+^ causes TCR signal suppression, resulting in human T cell exhaustion, decreased Ca^2+^ influx, proliferation, CD69 and CD25 expression both *in vitro and vivo* (120, 123). In addition, TRPM7, a non-selective Mg^2+^ channel, modulates Mg^2+^ concentration and T cell development and maturation (124, 125) and is fundamental for anti-tumor immunity (20, 126). Mouse TRPM7 deficiency did not affect acute uptake of Mg^2+^ or the maintenance of total cellular Mg^2+^ but suppresses T cell maturation (20, 49). Mechanistically, the binding between Mg^2+^ and metal-ion-dependent adhesion sites causes TCR signal stimulation, resulting in conformational changes in LFA-1, which is essential for T cell activation by mediating proximal and distal signaling activities, such as focal adhesion kinase phosphorylation and extracellular signal-regulated protein kinase 1/2 (ERK1/2) phosphorylation (50, 127–129). Lotscher et al. measured serum Mg^2+^ levels in 100 patients with leukemia and 67 patients with lung cancer and found that patients with low serum Mg^2+^ levels had lower survival rates to CAR-T therapy (128) (**Table 1**). In addition, activation and cytotoxicity are increased in intratumor CD8^+^ T cells and NK cells after intraperitoneal injection of MgCl_2_ (127) (**Figure 2**).

### 2.5 Potassium

K^+^ is an important inorganic electrolyte used to maintain normal physiological functions in the human body. K^+^ is involved in glucose, protein, and energy metabolism. K^+^ is the main cation in the cells and involved in maintaining osmotic pressure, myocardial function, and the acid-base balance of the intracellular and extracellular fluids (51, 130).

Similar to other cations, the balance of K^+^ within cells is finely controlled by several ion channels, including Kv1.3, KCa3.1, K2*p*3.1, K2*p*5.1, and K2*p*9.1 (131). Kv1.3 and KCa3.1 act as the main channels. The activation of Kv1.3- and KCa3.1-dependent membrane depolarization and release of Ca^2+^ and calmodulin, respectively, results in K^+^ efflux (52).

K^+^ has a crucial role in modulating T cell function and is associated with Ca^2+^ signaling (17, 132). TCR activation signals increase Ca^2+^ entry, resulting in intracellular Ca^2+^ release and membrane depolarization that causes Kv1.3 and KCa3.1 activation to increase K^+^ efflux, which in turn restores the membrane state, resulting in Ca^2+^ entry to induce NFAT expression, IL-2 release, T cell migratory capacity, and T cell subsets, including Th1, Th2, Th17, and Treg cells (133–135). In contrast, Ca^2+^ signaling is repressed if Kv1.3 and KCa3.1 are blocked upon TCR stimulation, indicating that the K^+^ gradient and Kv1.3 and KCa3.1 channels are essential for modulating T-cell activation (136). Moreover, reducing intracellular K^+^ via overexpression of KCa3.1 can increase the production of IFNγ in T cells to inhibit tumor growth and prolong survival, which is proved in patients with head and neck cancer (26, 53). In anti-tumor T-cell responses, large amounts of K^+^ produced by necrotizing tumor cells lead to a functional decline in CD8^+^ T cells owing to increased intracellular K^+^ affecting the Akt-mTOR pathway. Adenosine (Ado) and PD-1 signaling inhibit KCa3.1channel activity to limit K^+^ efflux leading to restrain the ability of CD8^+^ T cells from cancer patients (51) (**Figure 1**).

Another effect of K^+^ on T cells is the reprogramming of T cell metabolism. Higher extracellular K^+^ levels lead to autophagy and mitochondrial-based energy metabolism, causing changes in the expression of genes associated with activation, stemness, or exhaustion in T cells (137). In TILs isolated from B16 tumors, increasing extracellular K^+^ level induces central memory T cells, whereas reducing effector memory T cells and exhaustion markers, such as PD1, 2B4, and Tim-3 to enhance cancer immunotherapy. Hexokinase II also requires K^+^ as a cofactor to mediate anti-tumor immunity (137). In contrast, CD19-CAR-T cells express higher memory and lower effector genes after culturing in a medium containing a low glucose level and K^+^ (138) (**Table 1**). The anti-tumor effect of high K^+^ level was reported. The underlying mechanism was attributed to inhibition of tumor-associated macrophages (TAM), and the Kir2.1 channel was identified as the central regulator of TAM functional polarization in the high K^+^ TME (139) (**Figure 2**).

### 2.6 Iron

Fe^2+/3+^ has several biological functions in living organisms. Iron ions are important components of hemoglobin and myoglobin, which are used to maintain normal respiratory function (140). Second, iron ions participate in the catalytic reactions of various enzymes in the body, including fatty acid metabolism and oxidases. Iron ions also regulate various important metabolic processes, such as immune responses, neurotransmission, and DNA synthesis (141, 142).

Given the importance of Fe^2+^, its transport is finely regulated. Transferrin protein (Tf) is regarded as the primary transporter that forms a complex with Fe^2+^ and internalized by the transferrin receptor (CD71) (54, 143). In addition, DMT-1 and ZIP-8 are used to transport Fe^2+^ in a nonspecific manner (143).

T cell function and activation are impaired under conditions of defective Tf-receptors or decreased intracellular Fe^2+^ levels, such as reduced expression of CD25 and impaired to IL-2 receptor signaling (144). In contrast, Fe^2+^ supplementation in the culture medium can restore the biogenesis of T cell (145). Ferroptosis is an iron-dependent form of regulated cell death in both T and tumor cells. Fe^3+^ usually binds to transferrin in the form of trivalent iron, enters cells through transferrin channels, and is reduced to Fe^2 +^ by the metal reductase STEAP3, resulting in a Fenton’s reaction that releases reactive oxygen species (ROS) represented by hydroxyl radicals. Accumulated ROS-mediated peroxidation of membrane lipids leads to damage to cell function and ferroptosis. At present, ferroptosis is regarded as a method of inhibiting tumor growth, and a close correlation has been found between ferroptosis and anti-tumor immunity (55, 146). In a recent study, CD36 expression decreased cytokine release and inhibited the anti-tumor activity of B16 tumor-infiltrating CD8^+^ T cells owing to CD36-mediated lipid peroxidation and ferroptosis (147). In contrast, CD8^+^ T cells can induce tumor ferroptosis via lipid peroxide accumulation triggered by CD8^+^ T cells-secreted IFNγ to increase STAT1 and suppress cystine/glutamate antiporter system Xc in B16 beard mouse model (148). Peng Liao et al. found that arachidonic acid and IFN-γ secreted by CD8^+^ T cells in the TME can induce MC38 and B16F10 cell ferroptosis (149) (**Figure 1**).

According to a previous report, Fe^2+^ is released by other immune cells, such as TAMs and tumor-associated neutrophils (TANs), to sustain cancer progression (150). Fe^2+^ transporters and metabolism play an essential role in macrophage polarization; for example, M1 macrophages tend to reduce the level of Fe^2+^, whereas M2 macrophages are inclined to recirculate free Fe^2+^ (151). Kim et al. reported that myeloid-derived immunosuppressive cells (PMN-MDSCs) in the TME spontaneously induce oxidized lipid release, causing repression of T cell activity. In mice with normal immune function, suppressing ferroptosis inhibits the immunosuppressive activity of PMN-MDSCs and can be combined with immune checkpoint inhibitors to inhibit tumor growth (152). In HCC, ferroptosis triggers tumor invasion of MDSCs via high mobility group protein B1 (153). In gastric cancer, NK cell levels are negatively correlated with the abundance of tumor-associated fibroblasts (CAF). CAF impair the anti-tumor ability of NK cells by inducing ferroptosis (154) (**Figure 2**).

Magnetic hyperthermia is a tumor therapy triggered by iron oxide nanoparticles, enabling an increase in antigen presentation, maturation of DCs, T lymphocyte recruitment, and a reduction in Tregs (155). Biocompatible PEG-coated ferrihydrite nanoparticles (PEG-Fns) mediate TAM polarization from M2 to M1 (156). Zhang et al. assembled quercetin and iron ions to prepare the nano-photosensitization agent, QFN. When QFN was decomposed, the released quercetin reduced programmed death ligand 1 (PD-L1) in tumor cells by decreasing the activation of JAK2 and STAT3 and reshaping the extracellular matrix (ECM) (157). In summary, these data indicate that iron plays a dual role in regulating immune and tumor cells.

### 2.7 Copper

Cu^+/2+^ is an important trace element and an essential component of many biochemical reactions. There are two types, Cu^+^ and Cu^2+^ in living organisms (158). Within cells, Cu^2+^ undergo redox reactions and metabolic pathways with other metabolites, such as cytochrome c redox reactions and intracellular respiration (159). In addition, Cu^2+^ is important components of many enzymatic reactions, such as intracellular oxygen site-catalyzed oxidation reactions. Finally, Cu^2+^ participates in the expression of many transcription factors that regulate various physiological reactions, such as nuclear factor-kappa B (NF-кB) and activator protein-1 (AP-1) (160–162).

Cu^2+^ deficiency or excess can lead to oxidative stress and toxicity. Intracellular Cu^2+^ is delivered to various cellular compartments to modulate intracellular processes (163). Consequently, Cu^2+^ homeostasis is closely modulated by solute carrier family 31 member 1/2 (CTR1/2) and SLC11A2/DMT1, as well as ATPase copper transporting α (ATP7A) and β (ATP7B), MT, glutathione (GSH), Cu^2+^ chaperone for superoxide dismutase (CCS), antioxidant protein 1 (Atox1), and cytochrome c oxidase copper chaperone 17 (COX17). Extracellular Cu^2+^ is reduced to Cu^+^ by binding to metal reductases of the STEAP family and is transported into cells via SLC31A1 or combined with Cu^2+^ chaperone protein antioxidant-1 (ATOX1) to the Golgi network via ATP7A/B (164, 165). Within the cytoplasm, excess Cu^2+^ is excreted via ATP7A/B and transferred from the Golgi apparatus to other organelles to mediate cellular copper homeostasis (56). CCS1 delivers Cu^2+^ to superoxide dismutase 1 (SOD1) in the cytoplasm (57, 166). Cu^+^ in the mitochondrial membrane is regulated by COX17 during transport (167). CCS and ATOX1 are important transporters of Cu^+^ into the nucleus (168, 169). Furthermore, elesclomol, ATN-224, tetrathiomolybdate, disulfiram (DSF), pyrithione, and chloroquine are considered copper ionophores (58).

Cu^2+^ potentially is involved in the activation and regulation of T cells. Cu^2+^ deficiency impairs CTL capacity to kill target cells and reduces T cell proliferation both *in vitro* and *in vivo* (170, 171). Bala et al. found that the infiltration of CD4^+^ and CD8^+^ T cells decreased in splenic monocytes from copper-deficient rats (59). Both the protein and mRNA expression of IL-2 are reversibly decreased in human T lymphocytes, as well as superoxide anion production by neutrophils after Cu^2+^ deficiency (172–174). Moreover, the effect of Cu^2+^ on helper T cells is consistent with CTLs including their proliferation and function (175, 176). Voli et al. demonstrated that intracellular Cu^2+^ regulates PD-L1 expression to inhibit the function of T lymphocytes in brain tumors. Further exploration revealed that copper chelators decrease PD-L1 expression by suppressing the epidermal growth factor receptor (EGFR) and transcriptional activator protein (STAT) signaling pathways to increase the number of T lymphocytes and NK cells (177) (**Figure 1**).

Like other ions, Cu^2+^ also regulate other immune cells. Chatterjee et al. demonstrated that CuNG (a copper chelate) reprogrammed TAMs from M2 to M1, activating the T cell function (178, 179). Blood neutrophils are reduced in copper deficiency by decreasing the superoxide anions (180) (**Table 1**).

Cuproptosis is a unique mode of cell death and first reported by Tsvetkov et al. in 2022 (181–184). The classical mechanism of cuproptosis involves the binding of elesclomol to extracellular Cu^2+^ and its transport to the cellular compartment. Ferredoxin 1 (FDX1) reduces Cu^2+^ to Cu^+^ and inhibits iron-sulfur protein cluster (Fe-S) synthesis. Together, these abnormal processes lead to protein toxicity, ultimately leading to cell death. Wang et al. showed that chimeric antigen receptor T (CAR T) cells were reprogrammed by exposing DSF/Cu plus ionizing irradiation (IR). This method can reverse the immunosuppressive TME and increase the anti-tumor function of CAR-T cells (185, 186).

A nanoparticle drug, NP@ESCu, was developed to induce cancer cell apoptosis and simultaneously reprogram the immunosuppressive TME. When combined with αPD-L1 blockade, the therapeutic effect on cancer was obviously increased, which contributes to a broad range of clinical applications (187). BSO-CAT@MOF-199 @DDM (BCMD) nanomediated copper proliferation can induce immunogenic cell death (ICD) and enhance DC activation and T cell infiltration (188). In summary, although copper plays a key role in the activity of various enzymes, cytotoxicity can occur at higher concentrations.

## 3 Conclusion

Tumor immunotherapy has become one of the most promising treatment methods besides surgery, radiotherapy, and chemotherapy. However, tumor immunotherapy is influenced by various factors. The metabolism of metal ions in the tumor microenvironment, particularly their association with anti-tumor immunity, has been well-demonstrated. Metal ions can improve the tumor microenvironment and promote the activation and proliferation of immune cells. On the one hand, they activate the innate immune system by enhancing the presentation of DCs and the cytotoxicity of macrophages and NK cells to augment their anti-tumor effects. On the other hand, metal ions can also stimulate the activation and proliferation of adaptive immune cells, especially T cells that recognize and kill tumors to improve the effectiveness of tumor immunotherapy. With the continuous deepening of research, it has been found that metal ions can also directly induce tumor cell death, such as ferroptosis and cuproptosis, which mainly induce tumor cell death by changing their metabolic mode. Finally, metal ions are also used to prepare nanomedicines that can directly induce tumor death or exert anti-tumor effects by loading anti-tumor drugs and small RNAs to stimulate the immune cells. Although the application of metal ions appears to be a promising method for improving the efficacy of tumor immunotherapy based on existing results, making it a potential immune system agonist requires more attention regarding its safety.

## Author contributions

YG: Formal Analysis, Investigation, Resources, Supervision, Writing – original draft, Writing – review & editing. SL: Funding acquisition, Methodology, Project administration, Supervision, Writing – original draft. YH: Methodology, Project administration, Resources, Supervision, Writing – original draft. FL: Methodology, Supervision, Writing – review & editing. YZ: Conceptualization, Funding acquisition, Supervision, Writing – review & editing.
